# BREATFEEDING AND CONSUMPTION OF SWEETENED FOODS

**DOI:** 10.1590/1984-0462/;2018;36;4;00019

**Published:** 2018

**Authors:** Lúcia Campos Pellanda

**Affiliations:** a Instituto de Cardiologia, Fundação Universitária de Cardiologia, Porto Alegre, RS, Brasil.

Porto Alegre, July 17, 2018.

To: Ruth Guinsburg, Editor of Revista Paulista de Pediatria

Dear Editor,

I would like to make some observations about the article “Influence of breastfeeding on consumption of sweetened beverages or foods,” by Adriana Passanha,^1^ published in this journal. The authors’ goal was to verify whether breastfeeding is associated with a lower prevalence of consumption of sweetened beverages or foods among infants. First, I commend the author and collaborators, as both breastfeeding and food introduction are extremely important to the development of infants.^2^ I believe that, if the authors had included the age group between zero and six months in the analysis, it would have enriched the work. We know that exclusive breastfeeding is recommended during this period; however, in clinical practice, the reality is different: babies with few weeks of life are given teas, juices, and other sweetened beverages. Since the study has a representative number of infants, including this age group would provide the reader with a comprehensive overview of the prevalence of consumption of sweetened beverages or foods. I also suggest adding the duration of exclusive breastfeeding, as it might not reflect the current recommendations.^3^ I believe that some of the data in Table 1 should have been discussed. For instance, infants assisted by the public health system consume almost 10% more sweetened foods or beverages that infants with private insurance, even though, currently, health professionals of the public system have access to complete and easy-to-understand materials about food introduction.^4^ According to the author’s findings, the orientation for mothers on food introduction for infants still has flaws. My question is: where are we failing as public health professionals? We know that only imparting knowledge is not enough to change the patient’s life.

Still on the discussion, the text would be clearer if the authors started it by addressing its main result: the prevalence of consumption of sweetened beverages and foods. This result was only mentioned in the fifth paragraph. Before that, the authors lingered on the prevalence of overweight in the population under study. Although very relevant,^5^ I believe these data should have been used to reinforce the importance of breastfeeding and proper food introduction. However, only after discussing the main finding. Lastly, I would have liked to read about the possibility of generalizing the results (external validity).

Dra. Lúcia Campos Pellanda

## References

[1] Passanha A, Benício MHD, Venâncio SI (2018). Influência do aleitamento materno sobre o consumo de bebidas ou
alimentos adoçados. Rev Paul Pediatr.

[2] World Health Organization/United Nations Children’s Fund (2014). Global nutrition targets 2025: breastfeeding policy brief
(WHO/NMH/NHD/14.7).

[3] Brasil. Ministério da Saúde. Secretaria de Atenção à Saúde.
Departamento de Atenção Básica (2015). Saúde da criança: nutrição infantil: aleitamento materno e alimentação
complementar.

[4] Brasil. Ministério da Saúde. Secretaria de Atenção à Saúde.
Departamento de Atenção Básica (2013). Dez passos para uma alimentação saudável: guia alimentar para crianças
menores de dois anos: um guia para o profissional da saúde na atenção
básica.

[5] World Health Organization (2014). lobal nutrition targets 2025: childhood overweight policy brief
(WHO/NMH/NHD/14.6).

