# Supplementary material for: BREATFEEDING AND CONSUMPTION OF SWEETENED FOODS
Source: Rev Paul Pediatr. 2018 Oct-Dec;36(4):524–5. doi: 10.1590/1984-0462/;2018;36;4;00019 (PMC6322799; doi:10.1590/1984-0462/;2018;36;4;00019)
Supplement: Related texts

# Reply: BREATFEEDING AND CONSUMPTION OF SWEETENED FOODS

**Authors:** Adriana Passanha

**Affiliations:** a Universidade de São Paulo, São Paulo, SP, Brasil.

Received on August 09, 2018.

São Paulo, August 9, 2018.

To: Dr. Lúcia Campos Pellanda

Dear Dr. Lúcia,

We would like to thank you for your interest in our work and the observations made about our article.

In answer to your questions, we chose to study only the age group corresponding to the second semester of life because the article belongs to a doctoral dissertation that included other studies conducted with this same population. We agree that it is important to know about the consumption of sweetened foods and beverages in the first semester of life, considering the early food introduction among infants, and we can say in advance that other studies are being carried out with infants in the age group between zero and six months.

Regarding the duration of exclusive breastfeeding, the methodology used did not allow us to obtain this information individually since the survey consisted of closed questions (yes/no) on the feeding of infants in the previous day. This option followed the recommendations of the World Health Organization (WHO) on how to carry out surveys on infant feeding practices, which indicate the collection of information about the current feeding status through questions on the food given to the infant in the previous 24 hours. This strategy aims to avoid the informant’s memory bias, especially with respect to the duration of exclusive breastfeeding.^1^

In relation to the discussion of the findings, we chose to address the influence of breastfeeding on the consumption of sweetened foods and beverages as the main focus of the session. Another article, at the design stage, will discuss in greater depth the influence of other variables on the food intake of infants, including a variable related to the location of outpatient follow-up.

Regarding the prevalence of consumption of sweetened foods and beverages being described only in the fifth paragraph, we understand the argument that such information could be at the beginning of the session, but we chose to discuss other aspects beforehand. Also, the reviewers, when analyzing the article, made no remarks about this issue. Concerning external validity, we pointed out in the discussion that the Survey on the Prevalence of Breastfeeding (*Pesquisa de Prevalência de Aleitamento Materno*) was conducted with a representative sample, which reinforces the external validity of the findings.

We thank you once more for the attention to this work and the contributions made, which certainly invite a careful reflection on how to tackle the early offer of sweetened foods and beverages to Brazilian children.

## References

[1] World Health Organization (2008). Indicators for assessing infant and young child feeding practices -
part 1: definitions.
